# Subvoxel Control of Fiber Orientation via Multidirectional Shearing in 3D Printing

**DOI:** 10.1002/advs.202511008

**Published:** 2025-10-29

**Authors:** Berin Šeta, Marco Brander, Michael Sandberg, Md. Tusher Mollah, Vipin Kumar, Jon Spangenberg

**Affiliations:** ^1^ Department of Civil and Mechanical Engineering Technical University of Denmark 2800 Kongens Lyngby Denmark; ^2^ Department of Mechanical and Production Engineering Aarhus University 8200 Aarhus N Denmark; ^3^ Manufacturing Science Division Oak Ridge National Laboratory TN 37932 USA

**Keywords:** 3D printing, anisotropy, fiber orientation, subvoxel control

## Abstract

Anisotropy, the characteristic of materials exhibiting different properties based on their direction, is widespread in nature. Conventional manufacturing techniques often fall short in recreating such complex anisotropy. While 3D printing allows for precise fiber deposition in anisotropic composites, previous studies have only achieved bulk reorientation of fibers at the voxel level in two dimensions. This limits the replication of localized 3D anisotropies found in natural materials. To address this, a novel 3D printing technique is presented that enables subvoxel control of fiber orientation in all three directions using multidirectional shearing via nozzle rotation and inclination. The fiber orientation control in these experimental tests is driven by a numerical model, enabling a fully digital approach to program microstructures within a strand. This programmability is demonstrated through mechanical and thermal tests, illustrating a localized and controllable response to external stimuli. Achieving such complex anisotropy holds potential across several fields, including wearables and biomedical implants, lightweight composite structures, and energy storage technologies such as batteries and supercapacitors.

## Graphical abstract

A novel 3D printing technique enables subvoxel control of fiber orientation in all three directions through multidirectional shearing using nozzle rotation and inclination. It allows localized and tunable mechanical and thermal responses through programmable microstructures within a strand. Driven by a numerical model, the method replicates complex anisotropy found in nature and opens new opportunities in composites, wearables, biomedical implants, and energy storage.

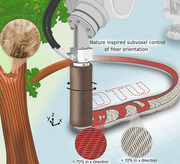

## 1 Introduction

**Figure 1:**
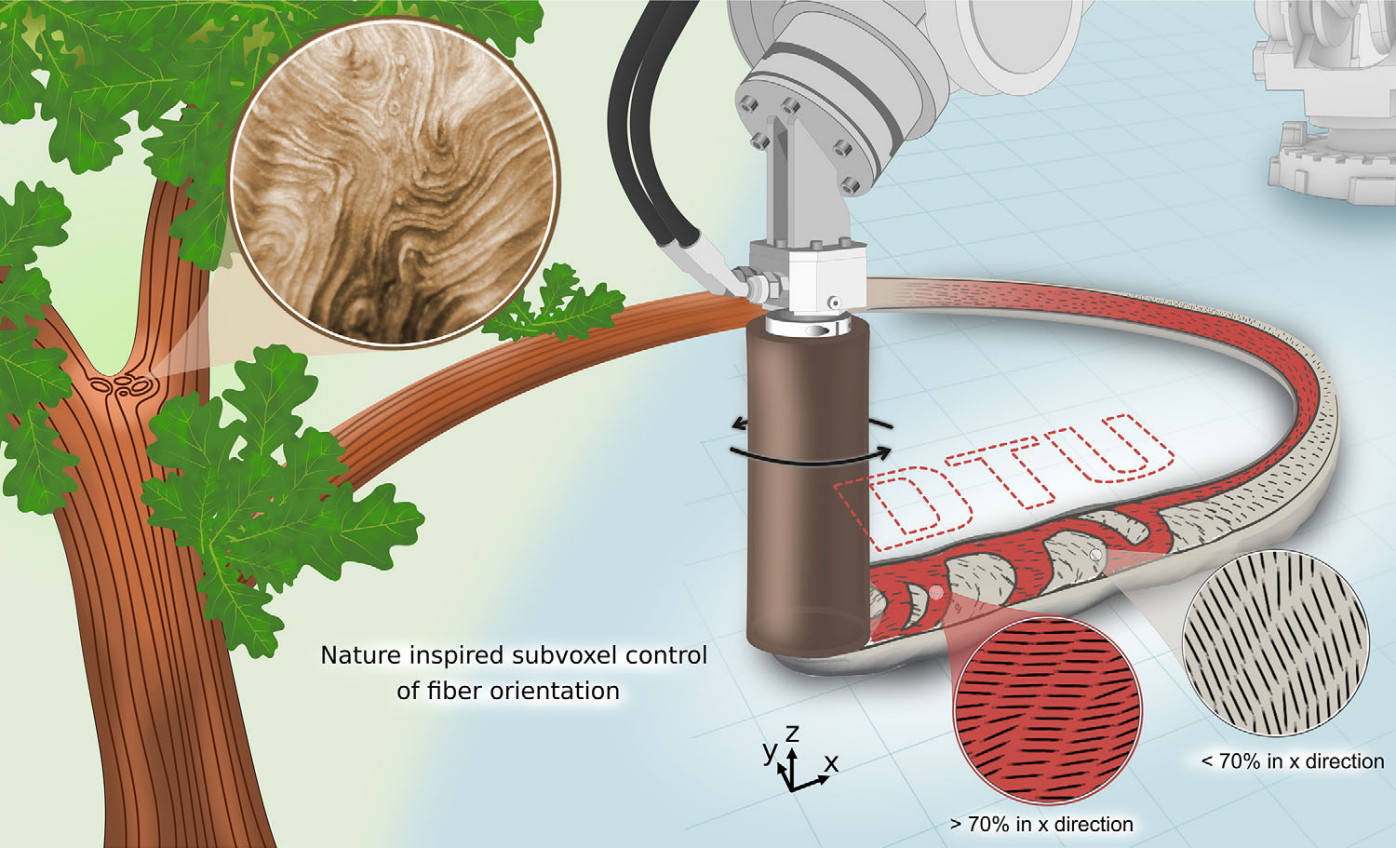
Example of the role of fibers in nature and a 3D printed fiber composite. Sketch of the interlocking mechanism found at a mature tree fork, where wood grains keep structural integrity and efficiently transport water by directing grains from source to sink.^[^ ^4^, ^33^ ^]^ Picture of a grain pattern in an ash wood showing an interlocking pattern.^[^ ^33^ ^]^ Fiber orientation control on a subvoxel level is achieved by rotational and translational motion of the nozzle. The fiber orientation reveals the “D T U” letters close to the top surface embedded into the microstructure of a single strand.

In nature, anisotropic properties are commonly found, enabling specific functions in organisms ‐ from the alignment of muscle cells in cardiac walls, which facilitates contraction^[^ ^1^, ^2^, ^3^ ^]^ to the grain structure in wood that provides directional mechanical strength and supports water transport^[^ ^4^ ^]^ (**Figure** **1**). Inspired by such natural systems, advances in 3D printing seek to replicate and control similar directional characteristics in engineered materials.^[^ ^5^, ^6^, ^7^, ^8^, ^9^, ^10^, ^11^ ^]^ Harnessing controlled anisotropy within printed materials opens possibilities for tailored mechanical, thermal, and electrical responses, a capability with far‐reaching applications across the construction, healthcare, energy and aerospace industries.^[^ ^12^, ^13^, ^14^, ^15^, ^16^, ^17^ ^]^

While 3D printed items do exhibit some anisotropic characteristics, these are generally considered limitations rather than features that can be deliberately controlled. The directional differences can stem not only from the printing process itself but also from the composite nature of the materials used, especially when multi‐material or fiber‐reinforced composites are involved. The anisotropy in fiber‐reinforced materials arises from the elongated nature of the fibers, which possess high aspect ratios ranging from tens to thousands. Such elongated particles influence the material properties^[^ ^18^, ^19^, ^20^, ^21^ ^]^ (mechanical, thermal, electrical, optical, acoustic) based on their orientations. Therefore, one of the key challenges in advanced 3D printing lies in achieving precise control over the alignment of fibers within composite materials. Since the inception of short‐fiber composite 3D printing, it has been widely accepted that fibers align primarily along the printing direction, dictated by the toolpath.^[^ ^22^, ^23^, ^24^, ^25^ ^]^ At the same time, it is important to recognize that not all applications necessarily benefit from strictly unidirectional alignment; in many cases, tailored multidirectional fiber orientations can enhance performance, for example, in components requiring multidirectional load‐bearing capacity or multifunctionality (Figure 1). Recently, the field has increasingly sought to decouple the fiber orientation from the toolpath, aiming to achieve subvoxel control ‐ a critical step for replicating the complex anisotropy observed in natural materials.

A few research groups have explored methods to control fiber alignment and decouple it from the toolpath, using techniques such as applying external magnetic forces,^[^ ^26^, ^27^, ^28^ ^]^ implementing active movements through rotation and flexible nozzles,^[^ ^29^, ^30^ ^]^ as well as through passive control by changing nozzle designs.^[^ ^31^ ^]^ While these approaches have succeeded in altering fiber orientation, the most pronounced re‐orientation effects were achieved with rotating nozzles and magnetic fields.^[^ ^27^, ^29^ ^]^ However, control using the magnetic field is limited by material compatibility, as it requires fibers that specifically respond to magnetic forces, restricting its applicability. Meanwhile, the rotating nozzle approach induces fiber re‐orientation through the process itself, making it effective with a broader range of particles with different morphology and type, such as graphene flakes or fibers. However, while state‐of‐the‐art rotational nozzle methods have demonstrated promising control over bulk fiber alignment,^[^ ^29^, ^32^ ^]^ they remain limited to voxel‐level adjustments, where the orientation is consistent across individual strands. Moreover, existing approaches rely solely on planar rotational printing and microscopic nozzles, which limits the fiber orientation to two directions and their scalability.

Our rotational printing approach advances this by achieving subvoxel control ‐ similar to the micro‐scale organization seen in natural systems ‐ allowing precise manipulation within single strands. This level of control, which has not been demonstrated in other printing systems to date, can be achieved through our novel oscillatory printing approach, which changes the direction of rotation during printing. Moreover, we introduce inclined rotational printing, enabling significant fiber alignment in the critical z‐direction as well, overcoming the previous limitation to 2D planar re‐orientation. With subvoxel control in mind, our approach excels in big‐area additive manufacturing, where tailored microstructures highly benefit from being achieved within a single or few strands, though it is adaptable to any scale. Finally, one of the key novelties of this work lies in its integration of experimental findings with a numerical model that simulates complex nozzle movements and accurately predicts fiber alignment in three dimensions. This model is the first in the literature capable of dynamically adjusting printing parameters, including rotation and inclination, to enable programmable fiber orientations. These novel capabilities open pathways to engineer materials with complex anisotropy (see Figure 1, D T U pattern) using 3D printing, potentially replicating the sophisticated structures seen in nature.

## 2 Results

### 2.1 Re‐Orientation Mechanism

**Figure 2:**
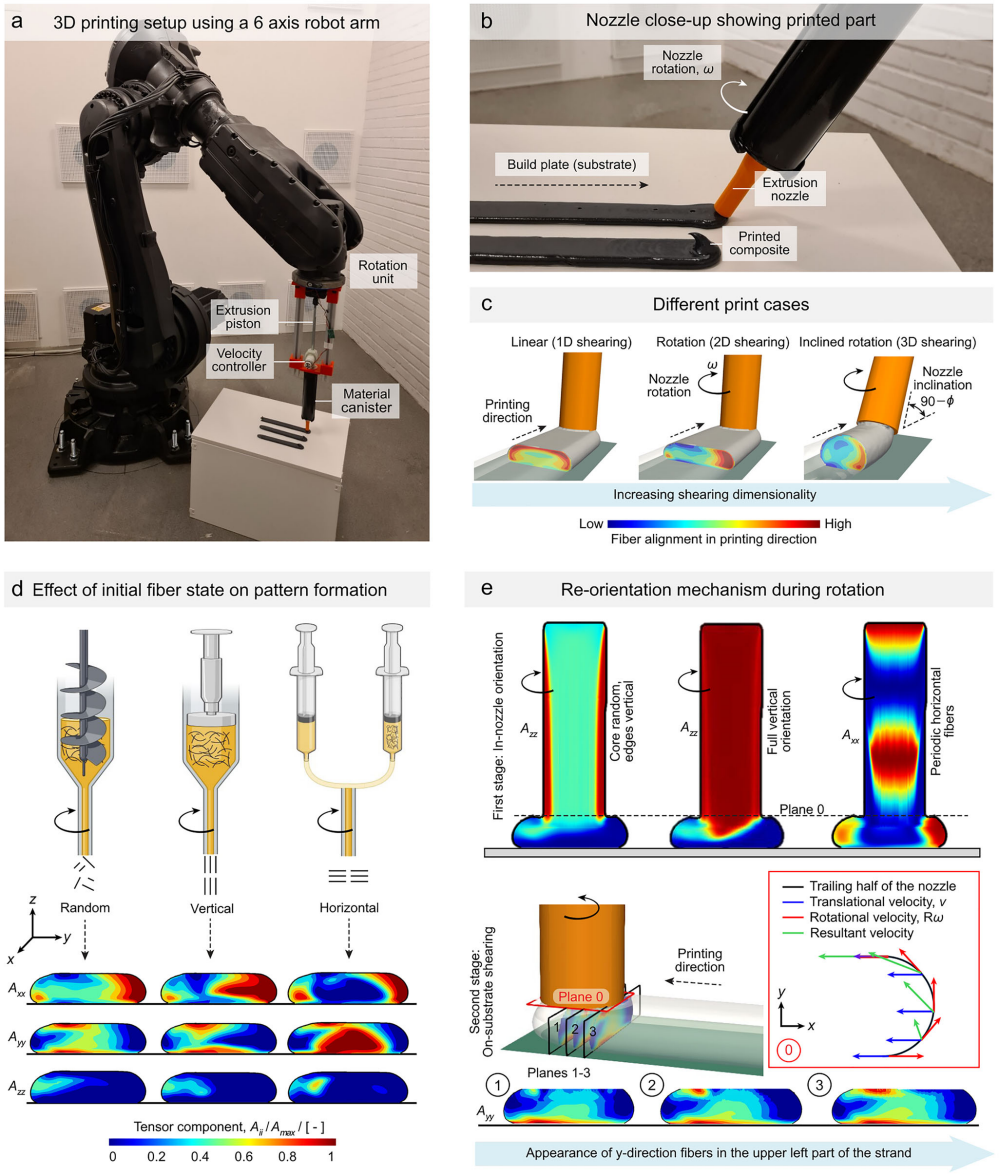
Experimental setup with three different modes of printing and fiber re‐orientation mechanisms during 3D printing. A) Image of robot arm during vertical extrusion with highlighted components of the system. B) Close‐up of the extrusion system with inclined nozzle. C) CFD simulations of the three modes of printing, Linear printing (1D shearing), rotation without inclination (2D shearing), and rotation with inclination (3D shearing). D) Different extrusion systems result in different patterns during the rotation. E) Two‐stage re‐orientation process. The first stage is contained to the nozzle and the fiber orientation depends on the extrusion system from d. The second stage is the re‐orientation mechanism when the tip of the nozzle shears over the material during its deposition. This influences the zone close to the top of the strand.

The 3D shearing phenomena that controls the fiber orientation in the material extrusion additive manufacturing (MEX‐AM) process is presented both experimentally by use of a six‐axis robot arm with accompanying extrusion piston (**Figure** **2**a,b) and numerically via a Computational Fluid Dynamics (CFD) model (Figure 2c). The control was derived from the fact that shearing plays a significant role in fiber orientation during 3D printing, whether inside the nozzle or at its tip. The thermoset components used for experimental printing were Sikaforce 818 L7 and Sikaforce 050, mixed with 0.1 mm long milled carbon fibers (more details are available in the Methods, 3D printing subsection). The fiber orientation is described using a second‐rank orientation tensor **A**, in which the diagonal elements (**A_xx_**, **A_yy_**, **A_zz_**) represent the fraction of fibers oriented in each principal direction^[^ ^34^ ^]^ (see Experimental Section, Microscopy subsection for more details, Figure S1, Supporting Information). The same tensor approach to assess fiber orientation was used in the numerical simulations.

Without any nozzle rotation, the fiber orientation in the cross‐section of the deposited strand resembled a surface‐core configuration, as depicted in Figure 2c. The fibers that align in the printing direction predominantly enveloped the strand's surface, especially along the sides and top.^[^ ^35^, ^36^ ^]^ This arose from 1D shearing inside the nozzle, aligning fibers near the nozzle walls in the direction of the extrusion. Additionally, when no rotation was applied, the nozzle tip created a 1D shearing pattern on the top of the strand after deposition, further enhancing the fiber alignment locally. In the core region, which is the central zone of the strand cross‐section, the orientation was very dependent on the fiber orientation at the nozzle inlet. Moreover, the rheological behavior can have an impact on fiber orientation,^[^ ^37^ ^]^ as it influences the flow profile during extrusion and the morphology of the strand after deposition.^[^ ^38^ ^]^

Introducing nozzle rotation and inclination altered this surface‐core configuration and was used to control the fiber orientation through 2D and 3D shearing, respectively (Figure 2c). The observed effect of rotation on the deposited strand for different extrusion systems (i.e., different inlet conditions) and their two‐stage re‐orientation mechanism are shown in Figure 2d,e. The first stage related to the shearing experienced inside the nozzle, while the second involved on‐substrate shearing. For all three extrusion systems (Figure 2d), a noticeable change occurred in the top part of the strand, where the shearing effect from the rotating nozzle tip was highest. Two separate zones were formed; one side shows orientation predominantly in a horizontal (*y*‐) direction, while the other displayed orientation in the printing (*x*‐) direction. The horizontal fibers were located in the areas where the resultant velocity of the nozzle (a combination of rotational and printing velocities) was primarily in the *y*‐direction (Figure 2e shows resultant velocity vectors in green). On the other hand, when the rotational and translational velocities were directionally aligned, the overall fiber alignment in the printing direction became even higher than in prints produced without rotation (1D versus 2D shearing in Figure 2c). When combining rotation and inclination, the additional shearing dimensionality was distributed between the y‐ and z‐planes (Figure 2c). As a result, fibers will become aligned in the z‐direction as well. These mechanisms control the formation of the separate fiber orientation zones and enable their deliberate spatial arrangement through changes in the rotation direction, allowing the zones to switch positions. This is crucial for achieving subvoxel control of the fiber orientation.

### 2.2 Experimental and Numerical Observations

**Figure 3:**
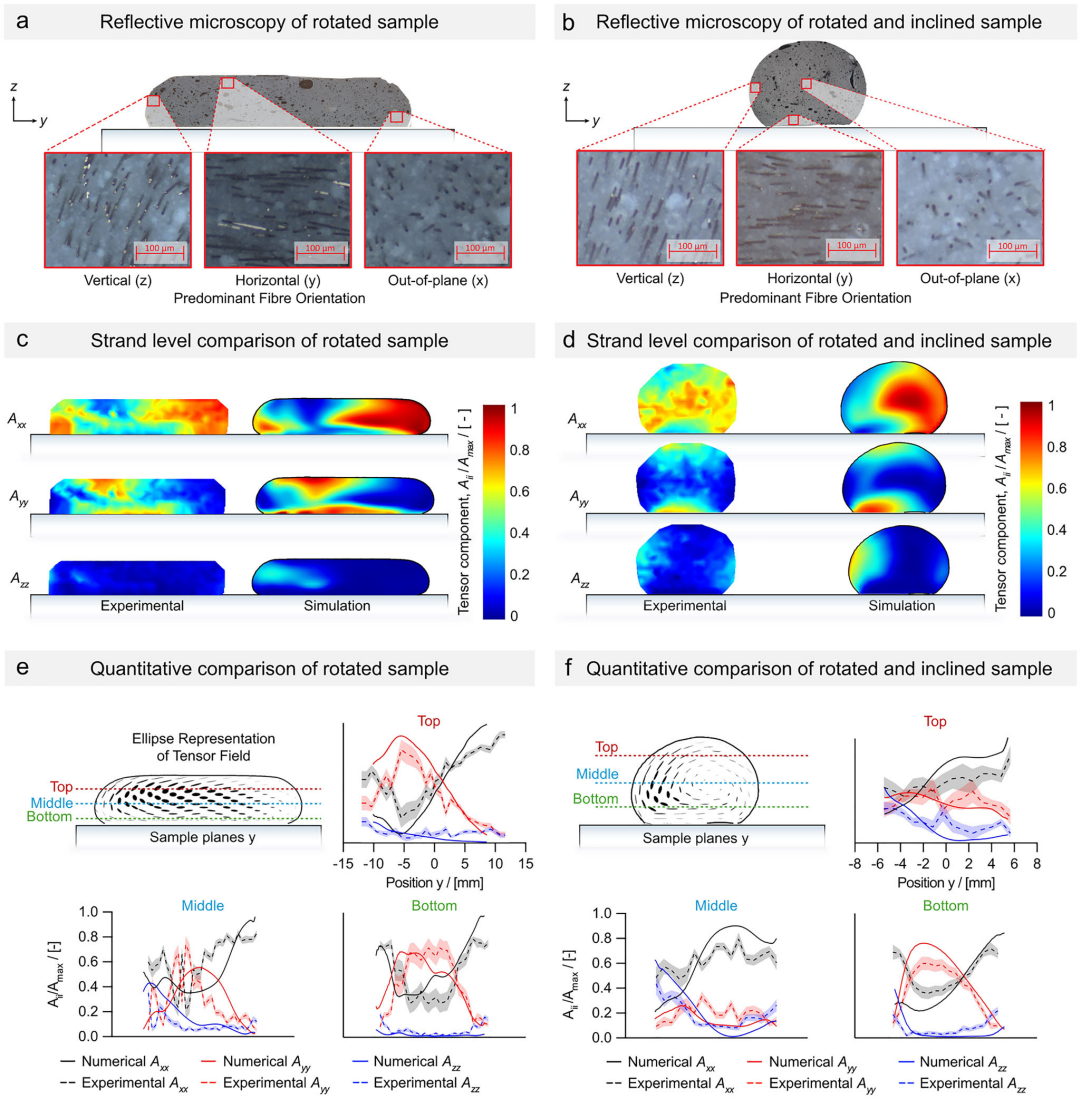
Experimental and numerical observation of fiber orientation in a strand cross‐section. A) Microscopy of sample without nozzle inclination. B) Microscopy of sample with rotation and a nozzle inclination of 30°. C) Comparison between numerical and experimental patterns for fiber orientation in all three directions in the non‐inclined case and D) the inclined case. E) Quantitative comparison for the non‐inclined case across three different lines in the strand cross‐section (top, mid, and bottom) with shaded 95% confidence interval and F) for the inclined case.

Two prints were prepared to demonstrate control of fiber orientation (**Figure** **3**); one with nozzle rotation and the other with 30° inclination as well as rotation. The fiber orientation was determined experimentally by cutting the printed strands and examining the cross‐sections using image analysis (Figure 3a,b, and Methods and materials, Microscopy subsection for more details, Figure 1, Supporting Information).

The resulting fiber orientations are shown in Figure 3c,d accompanied by the simulation results. There was a good quantitative agreement between experiments and simulations, (Figure 3e,f). For the prints without nozzle inclination, the cross‐sectional average of the fiber orientation (i.e., the cross‐sectional average of *A*_*ii*_) was found to be 56.8% in the *x*‐direction, 34.9% in the *y*‐direction, and 8.2% in the *z*‐direction. For the simulations, these numbers were 55.8%, 34.7%, and 9.5%, respectively.

The agreement motivated further numerical studies on how different rotational velocities affected the fiber orientation (Figure S2, Supporting Information) including the effects of different extrusion systems. The investigations considered two dimensionless parameters: 1) the velocity ratio given by the extrusion velocity (*u*) divided by the print speed (*v*), and 2) the normalized gap given by the height between the nozzle and substrate (*h*) divided by the nozzle diameter (*D*). Generally, the results showed similar trends for the two extrusion systems (i.e., screw and piston) when increasing the radius of the nozzle (*R*) and the angular velocity (ω) compared to the translational printing velocity (ω* = *R*ω/*v*); the fiber orientation toward the *y*‐direction was increased across the entire strand. Specifically for the piston extrusion system, the cross‐sectional average of **A_yy_** was raised from 7.9% (no rotation) to 44% (ω* = 3) (Figure S3, Supporting Information). The most significant change was observed at lower rotational velocities where, e.g. 35% of fibers were oriented in the *y*‐direction at ω* = 1, which was ≈5 times higher than without rotation.

Figures S2 and S3 (Supporting Information) also illustrate that it is not possible to completely decouple the fiber orientation from the printing direction, as some fibers always remain oriented in the printing direction on one side of the strand. The lack of further fiber de‐orientation beyond ω* = 2 can be explained by the slight offset of the deposited strands that were observed at higher rotation speeds due to the rotationally induced inertia. This offset shifted the y‐direction shear toward the strand's center, allowing fibers oriented in the x‐direction to reappear in the unsheared region on the left (Figure S3, Supporting Information). One can mitigate the offset by optimizing the printing parameters, which especially is important for multi‐layer printing. An example of how printing parameters affect the fiber orientation is demonstrated in the bottom part of the Figure S2 (Supporting Information), where the normalized gap is increased causing the x‐aligned fibers in the bottom left corner to disappear.

Finally, it is important to highlight that the re‐orientation mechanism yields consistent results across various fiber contents, ranging from a dilute regime (3%) to a concentrated regime (15%), cf. Figure S4 (Supporting Information). In this regard, the extent of anisotropy is influenced not only by the orientation of fibers but also by their concentration.

### 2.3 3D Shearing ‐ A Method to Introduce *z*‐Directional Fibers

Generally, limited fibers are oriented in the *z*‐direction during 3D printing with a vertical nozzle, whether it is rotating or not. This limits the process' capability to manufacture anisotropic components with attractive through‐thickness properties. However, by introducing a combination of nozzle rotation and inclination as demonstrated in (Figure 3d,f), it is apparent that more fibers can be oriented in the *z*‐direction as compared to the sample printed with rotation only. Here, our result showed that the proportion of the fibers in the *z*‐direction nearly doubled, increasing from 8.2% to 16%. The fraction of fibers aligned in the printing direction remained largely unchanged, from 56.8% to 57.1%, indicating that the increase in the *z*‐direction occurred at the expense of *y*‐direction oriented fibers (decreased from 34.9% to 27.2%). Similar observations were obtained using numerical analyses where 60% of fibers were aligned with the *x*‐direction, 24% with the *y*‐direction, and 16% with the *z*‐direction. The highest local fiber alignment in the *z*‐direction was observed at the left side of the strand, with a small presence also near the right edge. This occurred because the nozzle tip applied vertical shearing to the strand at its edges, while it sheared horizontally at the top, albeit shifted slightly to the left, similar to the observation for the non‐inclined samples.

The effect of the nozzle inclination angle and rotation velocity on fiber orientation was further studied through numerical simulations shown in Figures S5 and S6 (Supporting Information). The fiber orientation trends are similar for both angles, but as expected, ϕ = 30° resulted in more fibers aligning in the z‐direction due to the larger shearing component in the z‐plane. At high rotational velocities, the quantity of fibers oriented vertically exceeds those in the printing direction, which decreases from 92% to 23%. This indicated a shift from an almost unidirectional orientation to near‐isotropic properties. Further increases in the inclination angle did not lead to a higher degree of orientation in the *z*‐direction. Although the shear component in the *z*‐direction is larger (relatively to the cases with 15° and 30° inclination), the overall shear magnitude is reduced due to the weaker confinement of the fluid between the substrate and the nozzle tip. Moreover, at higher inclination angles, the uniformity of the strand becomes compromised, particularly at higher velocities, a phenomenon previously observed in multimaterial printing.^[^ ^11^ ^]^

### 2.4 Heat Transfer Anisotropy in a Single Rotated Strand

**Figure 4:**
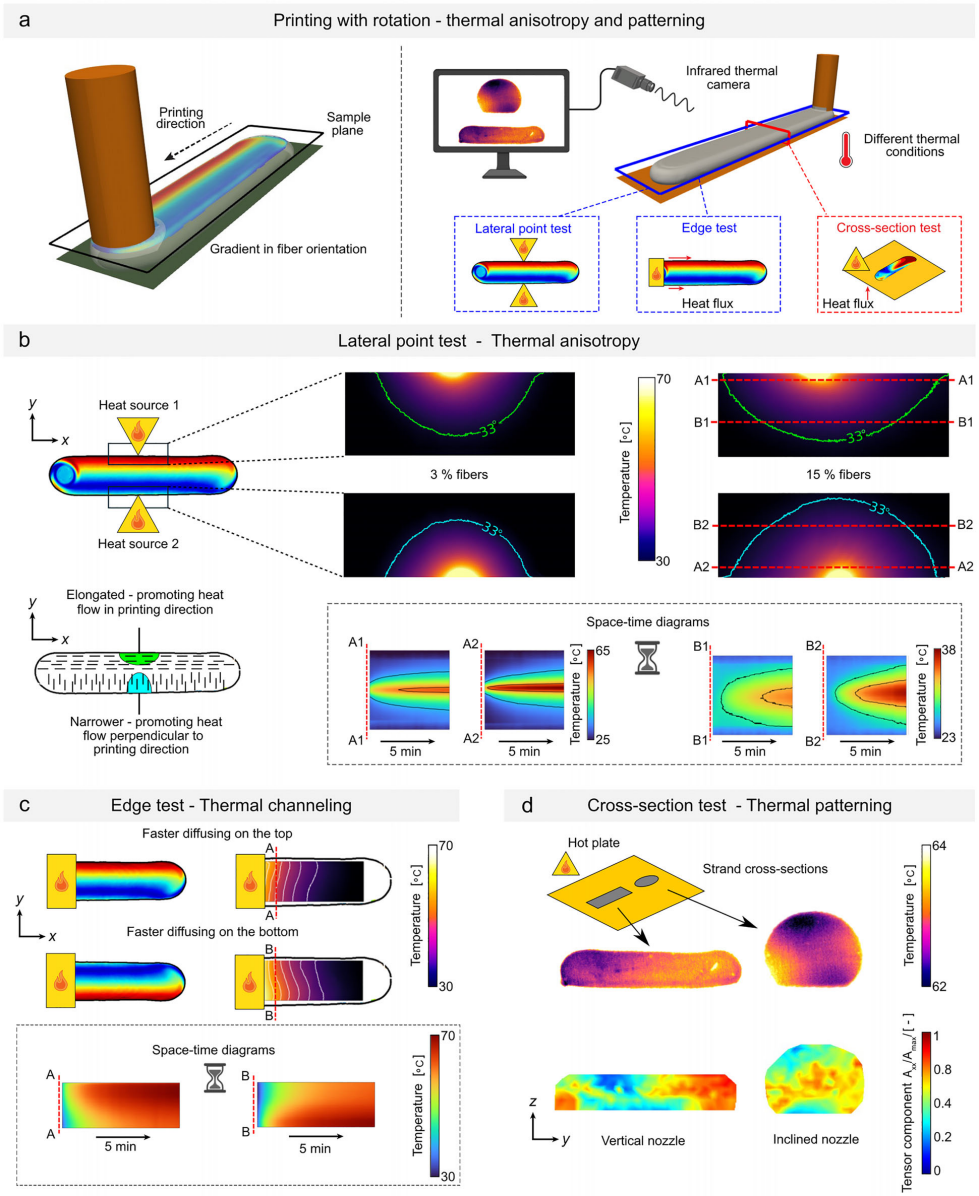
Heat anisotropy in rotated samples. A) Three types of conducted tests. B) Single‐point thermal tests applied to a sample produced with rotation. Experimentally observed temperature diffusion when the heat source is applied to two opposite lateral sides of a 3D‐printed strand. Time‐space diagrams illustrate the transient behavior along specific lines. C) Heat channeling through a rotated sample along its length, demonstrating that heat propagates faster on the side with highly aligned fibers. Space‐time diagrams are constructed for two cases with opposite rotation directions, showcasing the controllable transient behavior of heat channels. D) Thermal conductivity through the cross‐section of strands produced by nozzle rotation with and without inclination. Thin samples were placed on a heated bed, and their temperature was captured using a thermal camera to compare temperature patterns. High‐temperature zones (yellow‐red) correspond to regions with high fiber alignment perpendicular to the viewing plane.

Three tests were conducted to assess the impact of the local fiber orientation on the thermal properties of a single strand. In the first test (**Figure** **4**b), a sample was point‐heated from either side to observe the thermal conduction patterns in the *x*‐*y* plane (as viewed from above). In an isotropic material, the heat diffusion from a single point source forms a semi‐circular pattern. However, this behavior is altered by the presence of fibers that have a thermal conductivity orders of magnitude higher than the thermoset matrix. On the side with fibers aligned in the printing direction, heat diffusion adopts a more elongated elliptical shape along the *x*‐direction (indicated by the green curve, Figure 4b). This resulted in almost double the heat‐front propagation velocity in the *x*‐direction compared to the *y*‐direction for a fiber content of 15% (≈0.26 mm/s vs 0.13 mm/s), see Figure S7a (Supporting Information). On the side with fibers oriented across the strand's width, the diffusion pattern forms a narrower ellipse, showing higher maximum temperatures deeper into the material (represented by the cyan curve), compared to the previously described case. The heat‐front propagation velocities in this case are closer to each other (≈0.18 mm/s vs 0.14 mm/s), clearly indicating a different behavior from the previous case and exemplifying the effect of local anisotropy on heat transfer. This behavior has been demonstrated for two fiber contents, 3% and 15%, both yielding similar trends, albeit more prominent in the case with a larger amount of fibers. The higher fiber content enhanced heat transfer in both directions, as shown in Figure S7b (Supporting Information). After 5 min, the heat propagation front (defined at 33 °C) parallel to the printing direction reached 16.6 mm from the heat source for 15% fiber content, compared to 12 mm for 3%. This behavior is consistent with the higher conductivity expected from increased carbon fiber content. Furthermore, the results confirm that the re‐orientation mechanism remains effective at higher fiber fractions, as previously demonstrated by microscopy in Figure 4.

In the second experiment, the heat source is placed at the beginning of the rotated strand, with the expectation that heat will propagate faster on one side compared to the other. This behavior is observed, as shown in Figure 4c, where the heat front is shifted toward the highly oriented side. In addition to the skewed heat front, space‐time graphs across a single line perpendicular to the primary heat flux direction reveal that as long as the heat source maintains a higher temperature than the strand, heat continues to channel preferentially into the highly oriented region over time.

In the last test, thin slices of strands produced with and without nozzle inclination were placed on a heated bed to observe the thermal conduction patterns in the *y* − *z* plane (Figure 4d). For the non‐inclined sample, heat was conducted more rapidly on the right side than on the left, which reflected the local fiber orientation. In contrast, for the inclined sample, heat spread more swiftly along the edges and at the center, leaving cold spots at the top and bottom. The areas with higher temperatures correspond to the zones with fibers aligned in the printing direction (perpendicular to the viewing plane), illustrating that a single strand can exhibit varied thermal properties based on fiber orientation.

Although the anisotropic effect may not be drastic in the three experiments, we clearly demonstrate that it is still possible to achieve anisotropic heat transfer, heat channeling, and thermal patterning in a small space ‐ all of which are crucial for various applications. These include heat dissipation management (thermal anisotropy, Figure 4b), thermal shielding (thermal channeling, Figure 4c), and patterned or targeted hyperthermia (thermal patterning, Figure 4d). Finally, when the size of the printed part is scaled up and more complex patterns are designed, the effect of heat anisotropy will be even bigger.

### 2.5 Oscillatory Motion: A Way to Control Fibers Across the Length of the Strand

**Figure 5:**
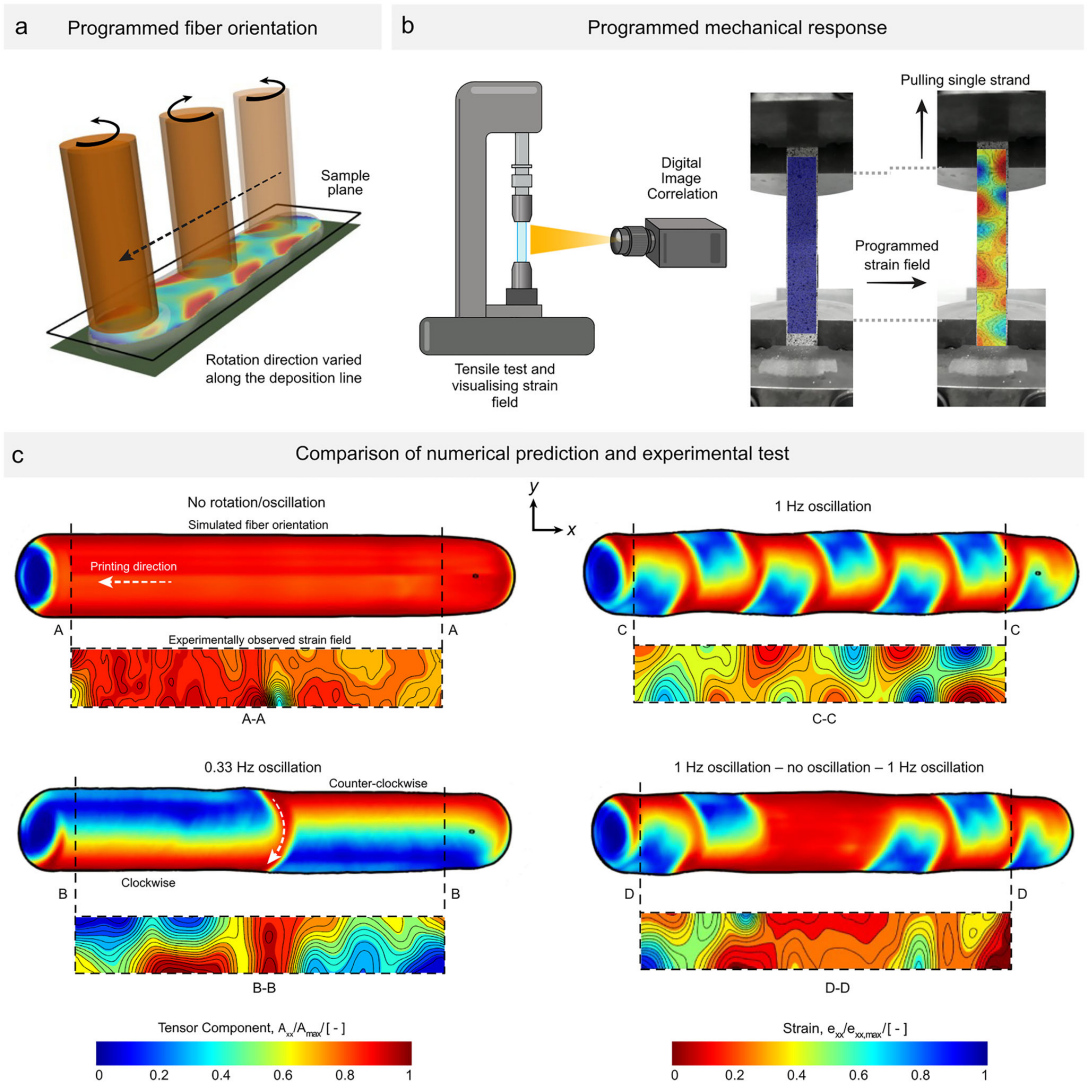
Programmed subvoxel mechanical anisotropy. A) Programmed mechanical anisotropy achieved through numerical prediction of fiber orientation. B) Tensile testing of experimental 3D‐printed samples with a programmed oscillation pattern, and accompanying strain fields analyzed using digital image correlation. C) Four different cases, each showing the predicted fiber orientation in a strand (top) and the tailored mechanical response through the strain field (bottom). The first sample was produced without rotation, while the second sample had an oscillation frequency of 0.33 Hz, resulting in a single transition of rotation direction within the observed area. The third sample, oscillating at 1 Hz, exhibited a checkerboard‐like pattern, and the fourth sample featured two isolated zones of oscillatory motion separated by a region without rotation.

Using a constant rotational velocity, with or without an inclined nozzle, samples exhibited a uniform fiber orientation along the strand. Altering the rotational velocity changed the size of the zones with fibers aligned in the printing direction, but these zones consistently remained on the same side of the strand. The ability to shift this zone to the opposite side adds another dimension to the fiber orientation control that can be achieved by reversing the rotation direction. In such case, the oscillation frequency (i.e., the rate of change in the rotation direction) is critical, as it dictates the length over which fibers with high orientation switch sides. This is shown in **Figure** **5**, presenting a top view of the strand, where depending on the oscillation frequency, the fiber orientation flipped sides. A transition zone was needed for this to develop, comparable in length to the nozzle wall thickness, where fibers across the width near the top remained aligned in the printing direction, as shearing forces from opposing rotations cancelled out.

The ability to control the velocity and direction of rotation opened the potential for creating even more intricate patterns, offering a high degree of control. An illustration of this is provided in Figure 1, where the letters D, T, and U were formed (the rotation profile required is shown in Supplementary Figure 8). This pattern was specific to a certain *x*‐*y* cross‐section of the strand, implying that cross‐sections at other *z* values might display different patterns. In this particular case, only the rotation parameters were changed. This involved changing the rotational velocity and acceleration to obtain a desired oscillation profile. The ability to adjust the fiber orientation at different sections of the strand significantly enhances the versatility and effectiveness of the 3D printing process in producing customized, structurally optimized components. One such example could be to reinforce the inner or outer part of a corner, inspired by an interlocking mechanism at a tree fork, as shown in Figure 9 (Supporting Information).

### 2.6 Mechanical Anisotropy in a Single Oscillatory Strand

Stands produced with different oscillation profiles were evaluated mechanically by tensile testing, cf. Figure 5. To ensure consistent testing conditions, the samples were cut and polished into rectangular shapes to achieve uniform thickness and width before conducting the tensile tests (further details can be found in the Methodology, Mechanical Tests section). Using digital image correlation, it was observed that the deformation patterns closely resembled the fiber orientation, showing the uneven strain distribution in the printing direction (*e*_*xx*_) across the strand.

The first sample had no rotation applied, and as expected, no visible pattern emerged apart from standard experimental non‐uniformities. The second sample was printed with an oscillation frequency of 0.33 Hz, leading to the alternating deposition of fibers aligned in the printing direction on the top side (from a top view) and then switching alignment to the bottom side. The observed pattern in the tensile test closely mimicked the programmed behavior, with high strain appearing in regions with lower fiber orientation in the printing direction. The third test increased the oscillation frequency to 1 Hz to examine the extent to which delineations between zones would persist without averaging out. At this frequency, six directional changes were programmed and observed during tensile testing, again, demonstrating the clear correspondence between the programmed and experimental patterns. Finally, an oscillatory motion was combined with no rotation to deliberately create distinct zones that controllably transition into each other. In this case, oscillation at 1 Hz with one directional change was followed by a no‐rotation phase, and then oscillation resumed. This produced a visible effect on the fiber orientation and strain field, where a uniform, strong zone was created in the middle, flanked by regions with non‐uniform (oscillatory) strain distributions. Interestingly, two out of three samples in this configuration broke almost simultaneously at two locations: the transition between the left oscillatory region and the no‐rotation region, and between the right oscillatory region and the no‐rotation region. This suggests that not only can the anisotropy be programmed, but the sites of crack nucleation ‐ and potentially their propagation ‐ can also be controlled.

## 3 Discussion

We have developed a novel 3D printing approach to achieve subvoxel control over fiber orientation in all three dimensions, utilizing multidirectional shearing. The control in our experimental tests was driven by a numerical model, the first of its kind, which allows complex nozzle movements during 3D printing, enabling a fully digital approach to microstructure design without relying on trial‐and‐error experimentation. The model was validated against experimental results for fiber orientation, demonstrating its accuracy in predicting the effects of different shearing modes.

Multidirectional shearing is achieved through three distinct modes: pure rotation of the nozzle, rotation with nozzle inclination, and oscillation. Pure rotation creates a zone of highly oriented fibers on one side of the strand, while the other side develops an expanding area of mostly horizontal fibers as the rotational velocity increases. Simultaneous rotation and inclination enable shearing in all three directions, forming a zone of vertical z‐fibers on one side of the strand. This is particularly relevant for 3D printing applications, as the z‐direction is often the weakest and achieving fibers oriented in this direction is especially challenging. Finally, oscillatory movement allows for the programmed placement of highly oriented zones along the length of the strand, enabling the creation of highly complex patterns, such as letters or nature‐inspired fiber formations found in tree forks.

Our methodology provides a wide range of control, allowing transitions from almost fully oriented fibers in the print direction to nearly isotropic. The shearing field can still partially be linked to the printing toolpath, and at high rotational speeds, a minor deposition offset may also occur due to inertia, though its impact on the strand morphology and fiber orientation is limited. Future work will focus on further refining these aspects by optimizing printing parameters, such as the gap height and extrusion speed, as well as the rheology of the material, to push fiber orientation control and deposition accuracy even further. In addition, our reorientation mechanism is process‐induced rather than material‐constrained, meaning it could be applied to various material matrices and fillers. A future study may, for example, examine how 2D graphene flakes introduce new forms of directional anisotropy.

As a result of our 3D printing technique, strands that appear identical to the naked eye can have entirely distinct internal microstructures. The impact of these programmed microstructures was demonstrated through a series of thermal and mechanical tests. The thermal tests confirmed directional heat transfer control via local fiber orientation, enabling the intentional creation of hot and cold spots and allowing for localized hypo‐ and hyperthermic conditions. This could enhance adaptive thermal regulation in smart wearables,^[^ ^39^ ^]^ improve thermal management in lithium batteries, and enable thermal shielding in space applications.^[^ ^40^, ^41^ ^]^ The mechanical tests revealed strain zones that closely matched the programmed fiber orientation pattern. This new‐found manufacturable mechanical anisotropy could be explored to design topology‐optimized composite structures that are not only theoretical optimal but also printable.^[^ ^42^ ^]^

Finally, while 3D printing has long been valued for its freedom in freeform shaping, performance has often been secondary. With our approach, this freedom extends beyond shape to include functionality, transforming 3D printing of smart composite structures across multiple scales, from the microscale to big‐area additive manufacturing. By achieving subvoxel control of anisotropy in a single printed strand, we gain a completely new level of freedom in programming anisotropy across multiple layers. Having in mind that subvoxel properties can be designed to respond to external stimuli, a new generation of complex and adaptive 4D‐printed architectures can be achieved.

## 4 Experimental Section

### 3D Printing

The thermoset components used for experimental printing were Sikaforce 818 L7 and Sikaforce 050, mixed with 3 Vol% 0.1 mm milled carbon fibers. Each test used a freshly mixed batch of printing material. First, 150 g of the Sikaforce 818 L7 was mixed with 8 − 55 grams of milled carbon fibers, depending on the fiber content of the test. The choice of carbon fibers was made based on the clear contrast between them and the otherwise white thermoset material. These were mixed for 90 seconds to ensure an even distribution. Afterward, 70 g of the hardener, Sikaforce 050, was added and mixed into the composition. Likewise, this was mixed thoroughly to ensure homogeneous distribution. The mix was then loaded into a 300 mL syringe canister, which was subsequently placed for 2 minutes in a DC‐26S vacuum chamber with an ECVP425 vacuum pump attached, to remove air bubbles.

The material canister was mounted in a test setup where a linear motor acted as a piston to force the material out of the syringe at set speeds. The discharge rate could be varied between 1 to 2.5 mm/s, equaling 2.26–5.65 mL/s extruded. This was in turn mounted on the head of an IRB6620 6‐axis robotic arm from ABB and linked up with the control unit of the robot arm. The extrusion setup was aligned with the rotational axis of the final joint of the robot arm. This permitted the joint to be used for nozzle rotation. The 5 other joints were used for global movement.

The prints were made using a circular nozzle of *D* = 12 mm that was 3D printed by a standard fused deposition modelling desktop printer, which allowed for extrusion speeds of 20–50 mm/s. The standard settings for the prints were a translational velocity equal to the extrusion of 20 mm/s, and a layer height of *D*/2 = 6 mm.

The movement was created in ABB's RobotStudio software, operating in the “RAPID” language. Here, each strand was designed using a number of sub‐points, where rotation was added by rotating the coordinates of each sub‐point by 80° around the sixth joint, as compared to the last point. The choice of rotating 80 degrees between each subpoint was made due to the interpretation of the software, which require rotations to be less than 90 degrees. Higher degrees of rotational speed were achieved by changing the distance between each subpoint, meaning the same amount of rotation needed to happen over a shorter travelled distance, while keeping a constant translation velocity. The rotational velocity had an upper limit, as the joint was only able to rotate at 180°/s. Each strand printed had a total length of 200 mm, in order to reach a steady state of extrusion and translation velocity.

Due to the size of the robot arm, during its movements, it has considerable momentum, which could lead to problems when changing directions of rotation at full velocity. Therefore, when the nozzle was 4.5° away from the point where the rotational direction would change, a “z‐factor” of 30 was implemented, allowing the joint to decelerate and accelerate around that point. The deceleration and acceleration around the shift point had a limited effect on prints with oscillations, while the samples with constant rotations were unaffected, except at the very ends of the strands.

### Microscopy

After printing, the samples were left to cure for 24 h to ensure further treatment would not deform them and thereby change the outcome of fiber orientations. Using a Struers Accotum‐50 rotary cutter, they were then cut into sections of 15 mm in length, and cast with the cross‐section facing downwards in cylindrical blocks in Struers EpoxyFit resin, with an EpoDye addition of 5 g/L.

The casted samples were polished in a Struers Rotopol‐21 polishing machine, using a rotational speed of 150 rpm, and a force of 5 N. First, the samples were polished at three different stages with silicon carbide sandpaper of grit 500, 1000, and 4000. Each stage was polished for 3 minutes. This ensured a smooth surface without any large scratches or grooves. Afterward, the samples were cleaned using ethanol and held for 10 s in an Emerson Branson 2800 ultrasound bath, to clean off particles and residue from previous polishing methods. The samples were then transferred to a Mol plate of woven fibers, where they were polished again for 3 min with a 3 µm diamond powder suspension, diluted in a Struers DP‐Lubricant. The cleaning method was repeated with alcohol and ultrasound bath before the final polishing on a NAP plate with free‐standing fibers was performed, this time using a 1 µm diamond powder suspension. After a final cleaning cycle, the samples were dried and sealed in a container to avoid scratches before the microscopy.

Once cast and polished, the samples were photographed using a Zeiss Axio Vert A1 microscope with an “Axiocam 305 color” camera attached. The choice of microscopy over µCT was motivated by the fact that the fiber diameter is very small (≈7 µm) compared to the overall strand size (≈2 cm in width). Consequently, in this case, covering the full strand cross‐section with µCT would be expensive and time‐consuming while offering limited benefits over microscopy for the fiber orientation estimation. Using a magnification lens of 100, the Zen‐core software was used to map the cross sections in pictures of 800x900 μm. The pictures were taken using bright light microscopy to make the fiber cross sections stand out against the matrix material. The pictures were then imported into a MATLAB script, which binarized them, identified the bright cross sections as fibers, and fitted them to ellipses via a least‐squares criterion.^[^ ^43^ ^]^ Using the orientation as well as the length of the minor and major axes of the ellipses, it was possible to evaluate the orientation of the fibers along the *x*‐, *y*‐, and *z*‐axis of the strand. The average fiber orientation among all cross sections in a picture was calculated and finally used to discretize the fiber orientation at 400 points of a strand, in which the orientations between points were interpolated linearly. The orientation was characterized through a second‐rank orientation tensor **A**, which was defined as a function of the angles θ and ϕ, cf. Figure S1 (Supporting Information):

(1) $$\begin{array}{ccc} A & = & \left[\begin{array}{ccc} A_{\mathrm{zz}} & A_{\mathrm{zy}} & A_{\mathrm{zx}}\\ A_{\mathrm{yz}} & A_{\mathrm{yy}} & A_{\mathrm{yx}}\\ A_{\mathrm{xz}} & A_{\mathrm{xy}} & A_{\mathrm{xx}} \end{array}\right]\\ & = & \left[\begin{array}{ccc} \sin^{2}\theta \cos^{2}\phi & \sin^{2}\theta \cos\phi \sin\phi & \sin\theta \cos\theta \cos\phi \\ \sin^{2}\theta \cos\phi \sin\phi & \sin^{2}\theta \sin^{2}\phi & \sin\theta \cos\theta \sin\phi \\ \sin\theta \cos\theta \cos\phi & \sin\theta \cos\theta \sin\phi & \cos^{2}\theta \end{array}\right] \end{array}$$

The fitted ellipses with corresponding minor (*m*) and major (*M*) axes, along with their projections on principal direction (*Z* and *Y*) were correlated with the ϕ and θ angles as follows:

(2) $$\begin{array}{cccc} \sin\phi & =\frac{Z}{M}\; & \cos\phi & =\frac{Y}{M}\\ \sin\theta & =\sqrt{1-\frac{m^{2}}{M^{2}}}\; & \cos\theta & =\frac{m}{M} \end{array}$$

As the viewing plane introduces bias, resulting in much higher chances of cutting through the fibers that were oriented perpendicular to the viewing plane of the sample,^[^ ^44^, ^45^, ^46^ ^]^ a weighting function *F*_*n*_ was introduced in Equation (3). This required correction in the experimental calculation of the orientation of the fibers as shown in Equation (4).

(3) $$F_{n}=\frac{1}{L\;\cos\theta_{n}}$$

(4) $$A=\frac{\sum_{n=1}^{N}A_{i}\;L\;F_{n}}{\sum_{n=1}^{N}L\;F_{n}}$$

where **A** is the bias‐corrected tensor and *L* is the length of the fibers that is assumed to be 100 µm. Uncertainties and potential errors related to microscopy as a technique for evaluating 3D fiber orientation are well documented and described elsewhere,^[^ ^44^ ^]^ and are applicable to this work as well.

### Thermal Measurements

In order to evaluate the impact of fibers on the thermal properties of the prints, heat sources from a FDM desktop printer were refurbished as a controllable source of heat. Here, the hot end of the print head was used as a point source, while the print bed functioned as a heated plate, allowing for even heating of a sample. A thermal paste Noctua N1‐H1 was used, in order to ensure thermal connection between the heat sources and the samples in question. Likewise, Styrofoam pieces were cut into shapes to contain the samples in Figure 4c, minimizing the effect of edge cooling of the plates. Data was acquired using a Optris PI thermal camera, while data processing was carried out with the Optris Pix Connect software.

Three different tests were performed. One was made by placing the point source of heat on the side of a full strand printed at a constant rotation velocity. All sides of the strand were insulated using Styrofoam, both to minimize cooling from the sides and to remove potential radiation from the heat source. The samples were heated with the point source at 90°C for 5 min, to see how the heat transferred into the strand. This test was performed with both a strand printed with a fiber content of 3 Vol% and 15 Vol%, in order to compare the effects. They were then left for 20 min to cool down to room temperature, before repeating the test on the opposite side of the strand. The second test was performed by using a flat heating source at the end of strands printed with a constant rotational speed. This was done in order to have a uniform heat input across the width of the strand, which allowed the two sides of the strand to diffuse heat at different rates. In the third test, thin cross sections of 2 mm in thickness were placed flatly on the heated bed set to 70°C. The temperature was captured directly from above, and the experiment ran until the samples and the heated plate had the same temperature. This allowed finding the difference in temperature increase across the cross‐section of the strand, and thus evaluating the difference in thermal conductivity.

### Mechanical Tests

Samples were tensile tested while using Direct Image Correlation (DIC) to measure the strain field. For this, samples of 250 mm were printed using the oscillation method. The printed samples were made into coupons by milling them slightly on the sides and the top, to ensure an even width and thickness of the samples. The strands were then sprayed with a chalk spray, giving them a fully white surface, before a speckled pattern was made with black paint. A servo‐press with a tensile testing feature was used to apply a constant deformation rate to the samples. The tensile test used a constant movement speed of 0.1 mm/s and captured 20 pictures per second, by a camera attached directly in front of the middle of the strand. Post‐processing of the data was done using the open‐source nCorr DIC program written in Matlab.^[^ ^47^ ^]^

### Computational Fluid Dynamics Model

The extrusion/deposition flow of the fiber‐reinforced polymer was simulated with CFD, using the open‐source software OpenFOAM (v2012, developed and maintained by ESI OpenCFD group). The finite volume method was applied to solve the governing equations (i.e., continuity and momentum). The volume‐of‐fluid (VoF) approach was employed to solve the multiphase flow, using the native MULES algorithm that is common for this family of InterFOAM solvers. This standard library was extended with the Advani‐Tucker tensor approach in order to predict the fiber orientation.^[^ ^48^ ^]^ The implemented model was two‐way coupled (flow‐fiber interactions), taking into account fiber volume fraction, fiber aspect ratio, and fiber‐fiber interactions. A hybrid closure approximation for the fourth rank tensor was used, as proposed by Advani and Tucker.^[^ ^49^ ^]^ The nozzle movement was treated by the overset methodology (overInterDyMFoam as a base solver), where the nozzle was treated as the overset mesh while the strand was deposited in the background mesh. The nozzle had six degrees of freedom, allowing simultaneous translational motion, rotation, and inclination. The rheological model used throughout the manuscript was the Bingham model, a typical material model applied to thermosets. Other modeling details, such as fiber‐fiber interactions and fiber‐flow coupling, together with governing equations are available in Šeta et al.^[^ ^35^ ^]^

## Conflict of Interest

The authors declare no conflict of interest.

## Supporting information

Supporting Information

## Data Availability

The data that support the findings of this study are available from the corresponding author upon reasonable request.
