# Supplementary material for: Subvoxel Control of Fiber Orientation via Multidirectional Shearing in 3D Printing
Source: Adv Sci (Weinh). 2025 Oct 29;13(9):e11008. doi: 10.1002/advs.202511008 (PMC12903997; doi:10.1002/advs.202511008)
Supplement: Supplementary file 1 — Supporting Information [file ADVS-13-e11008-s001.pdf]

Subvoxel control of fiber orientation via multidirectional shearing in 3D printing

Berin Šeta Marco Brander Michael Sandberg Md. Tusher Mollah Vipin Kumar Jon Spangenberg*

Supporting information

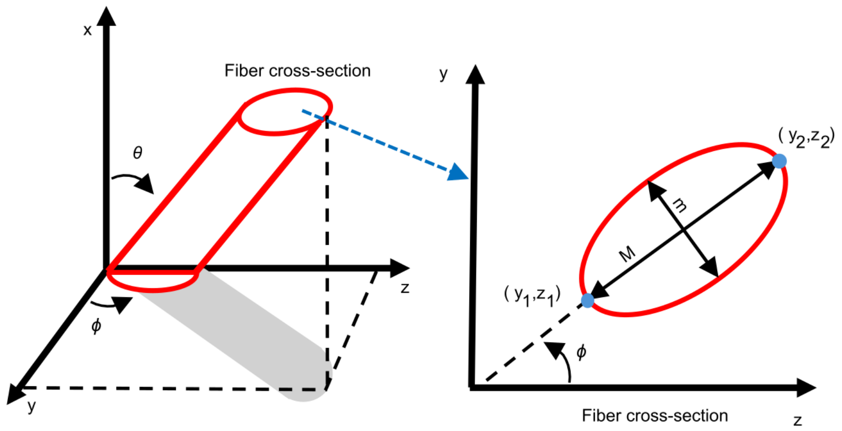

Supplementary Figure 1: Cross-section of the fiber and corresponding minor (m) and major (M) axes. The orientation tensor $\mathbf{A}$ is calculated based on the values of the angles ϕ and θ.

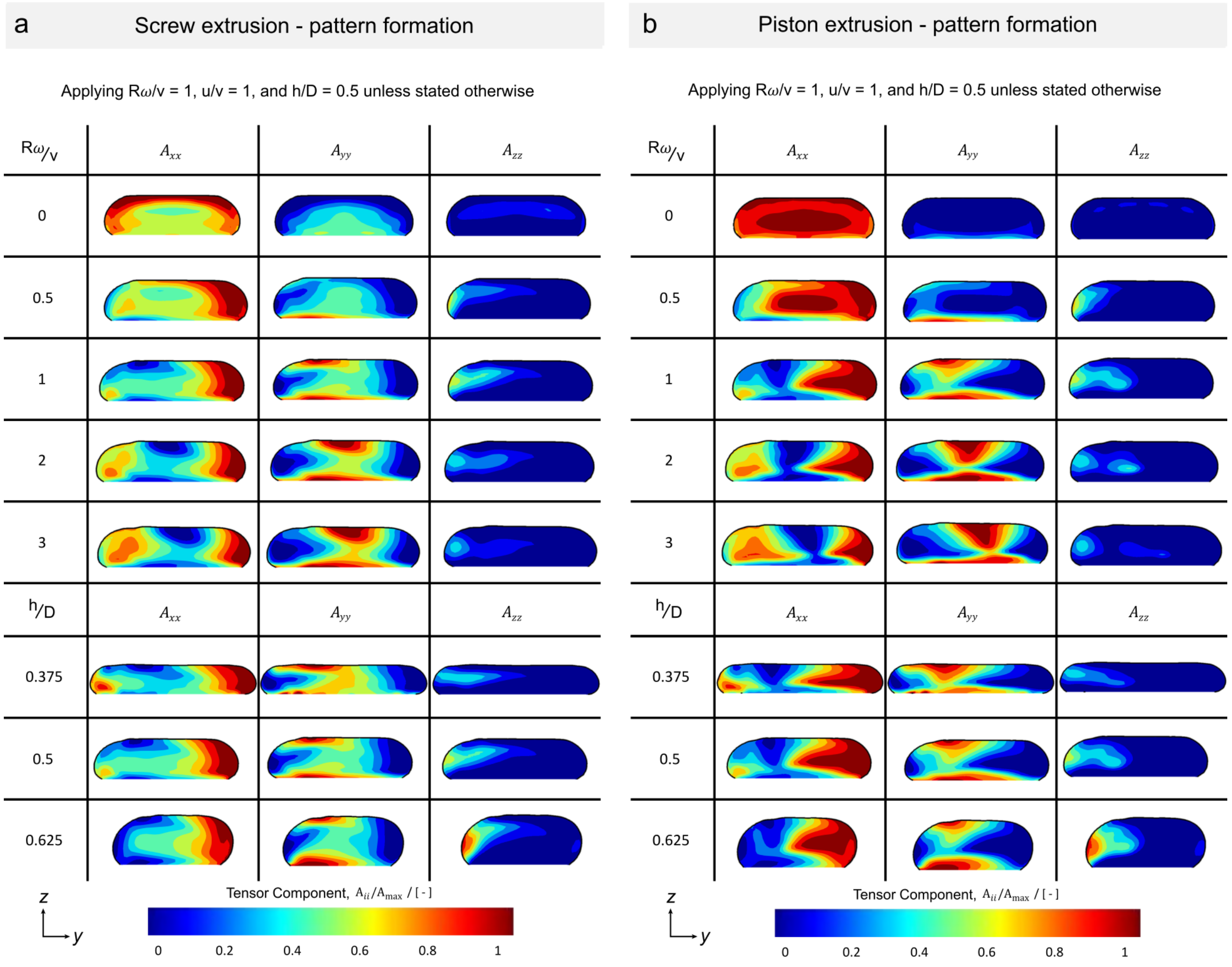

Supplementary Figure 2: Parametric study of fiber orientation pattern formation in, (**A**) Screw extrusion and, (**B**) Piston extrusion. The fiber configurations are shown for different dimensionless rotational velocities and normalized gaps. By changing the dimensionless rotational velocity, the strand morphology remains quite uniform while the orientation of fibers significantly changes. On the other hand, by changing the normalized gap, the strand morphology is altered, while patterns remain similar, albeit slightly distorted.

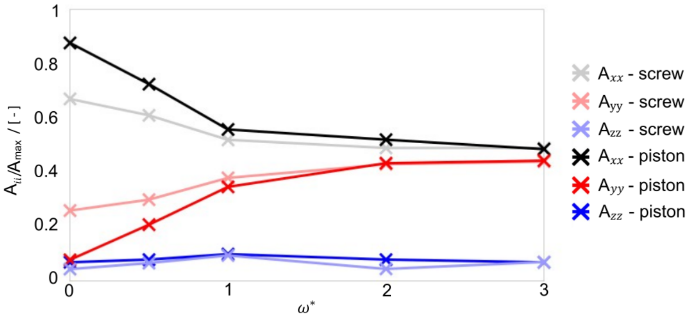

Supplementary Figure 3: Average fiber orientation in the cross-section of the strands produced with different dimensionless rotational velocities and a non-inclined nozzle (cf. Fig. 2).

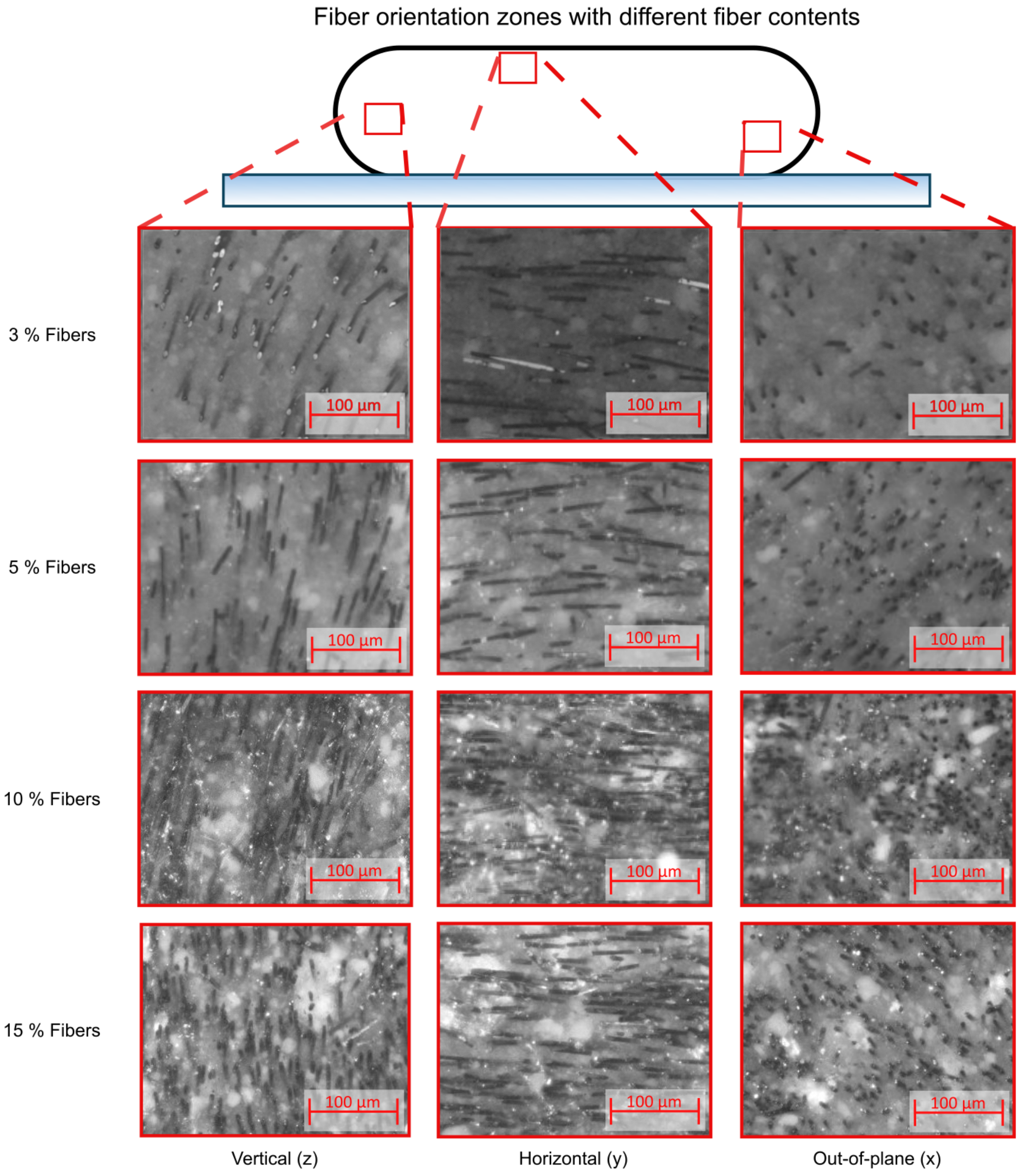

Supplementary Figure 4: Zones with predominantly oriented fibers in certain direction remain consistent across various fiber contents.

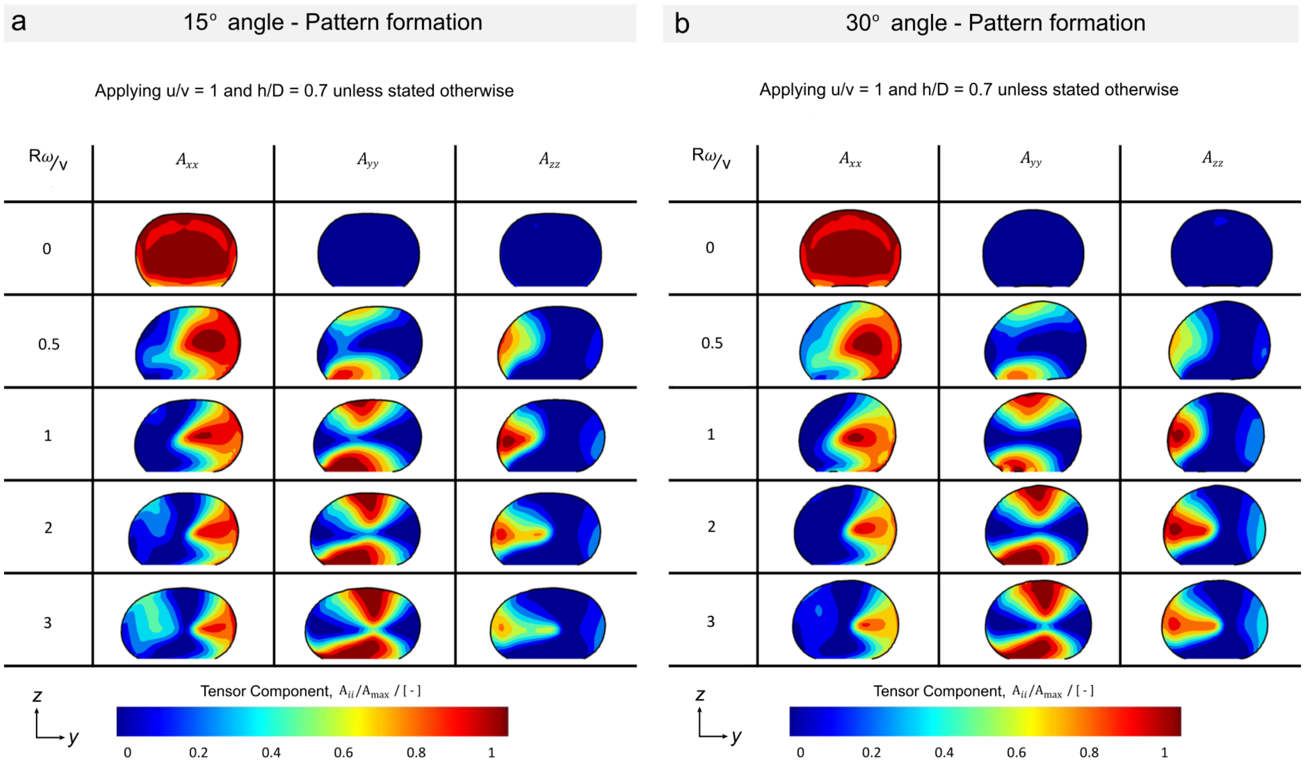

Supplementary Figure 5: Fiber orientation pattern at different dimensionless rotational velocities for two nozzle inclination angles: (**A**) 15° and, (**B**) 30°. A larger angle (30°) resulted in more fibers aligning in the vertical direction, especially for higher dimensionless rotational velocities. Generally, more fibers are oriented in the vertical direction when compared to the non-inclined results in Fig. 2.

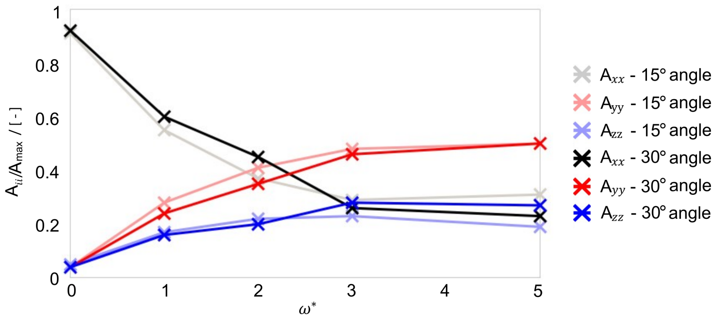

Supplementary Figure 6: Average fiber orientation in the cross-section of the strands produced with different dimensionless rotational velocities at two nozzle inclination angles (cf. Fig. 5).

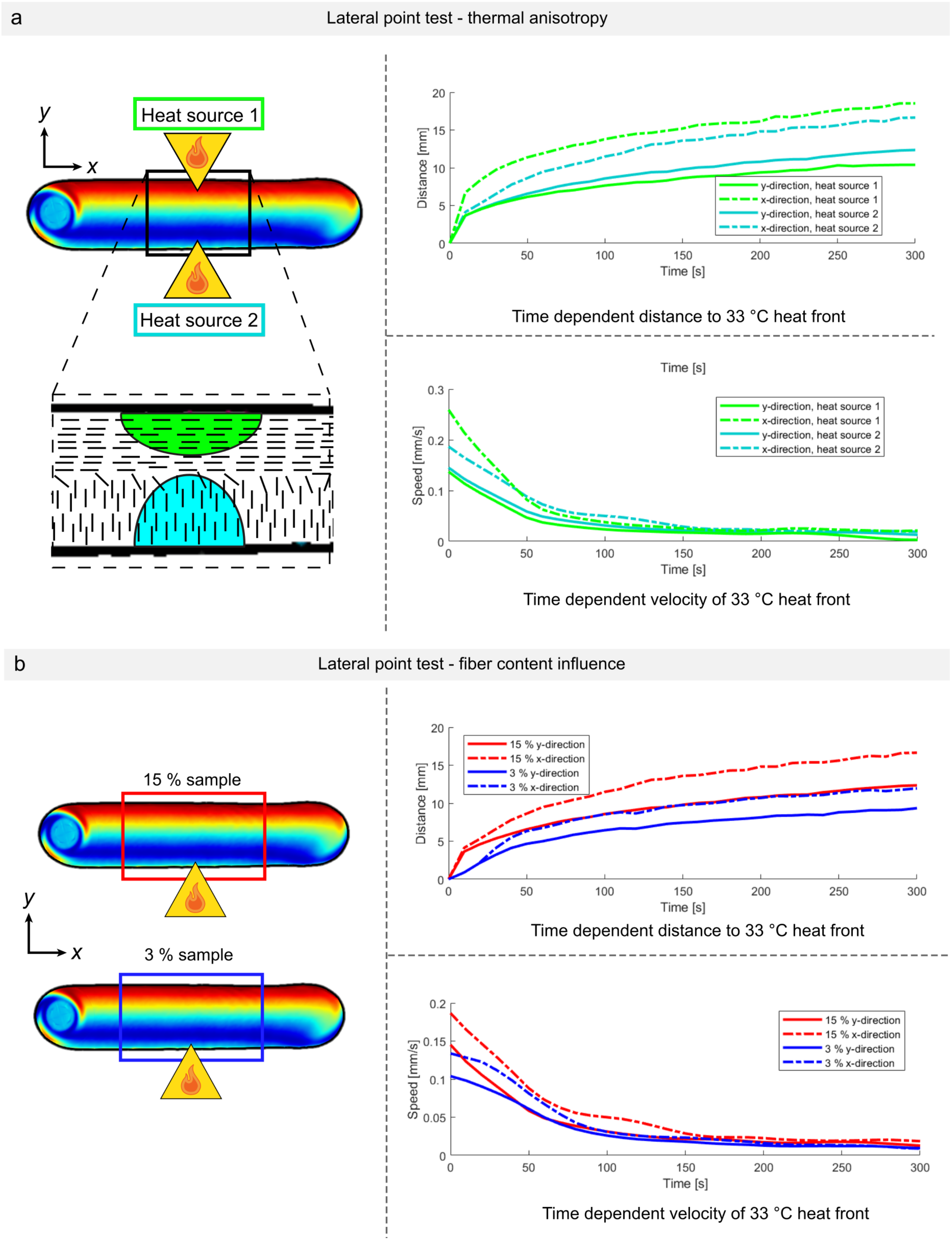

Supplementary Figure 7: Heat front propagation speed: (**A**) Opposite sides of the sample with 15% fibers, (**B**) Comparison between samples with 3% and 15% fibers.

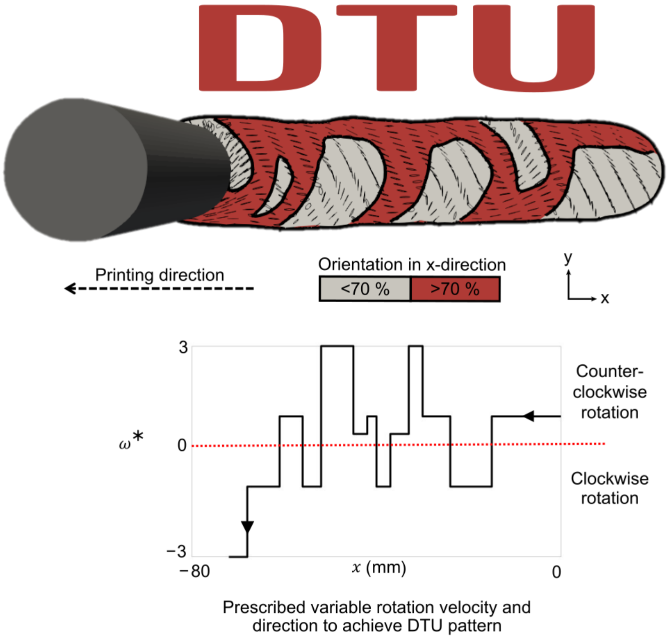

Supplementary Figure 8: Fiber pattern formation control on a subvoxel level exemplified by the formation of the letters D T U. It is obtained by varying the dimensionless rotational velocity. The signal for the ω^* is provided in the bottom part of the figure. The red regions represent highly aligned fibers in the printing direction ($>$ 70%), while the gray regions represent fibers with lower alignment in the printing direction ($<$ 70%).

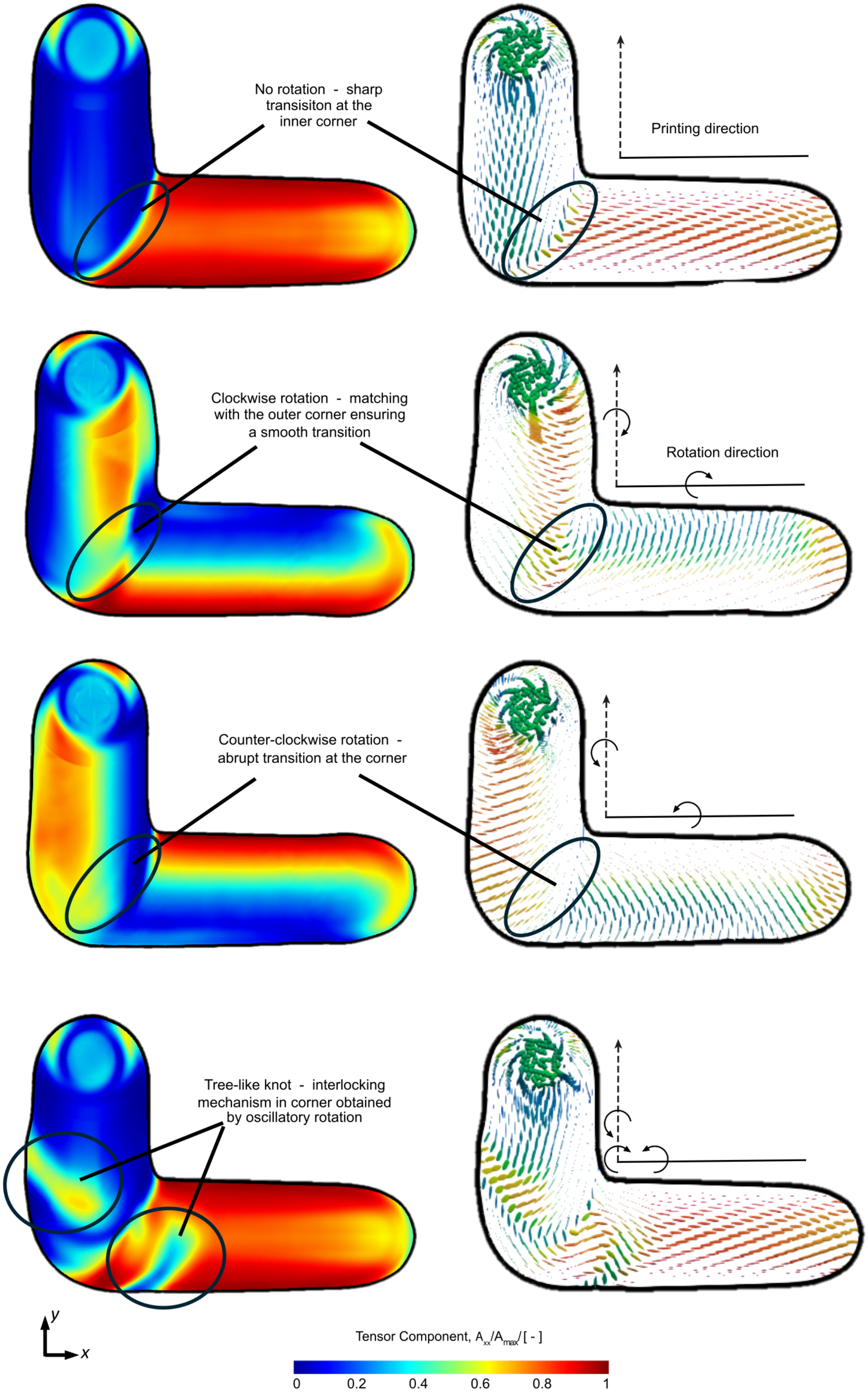

Supplementary Figure 9: Fiber patterns observed during corner printing with different strategies: no-rotation, clockwise rotation, counter-clockwise rotation, and customized rotation. The customized rotation produces an interlocking configuration.
